# Supplementary material for: Deciphering the Mechanism of Tolerance to Apple Replant Disease Using a Genetic Mapping Approach in a Malling 9 × M. × robusta 5 Population Identifies SNP Markers Linked to Candidate Genes
Source: Int J Mol Sci. 2023 Mar 27;24(7):6307. doi: 10.3390/ijms24076307 (PMC10094387; doi:10.3390/ijms24076307)
Supplement: Supplementary file 1 [file ijms-24-06307-s001.zip › ijms-2302833-supplementary/03_Supplemental_Material/Supplemental Figure S1_M9 map.pdf]

Supplemental Figure S1: High-density map for the ARD-susceptible rootstock M9 based on SNP and SSR markers. Linkage groups 1–17 represents the 17 chromosomes of *Malus domestica* GDDH13 v.1.1 reference genome.

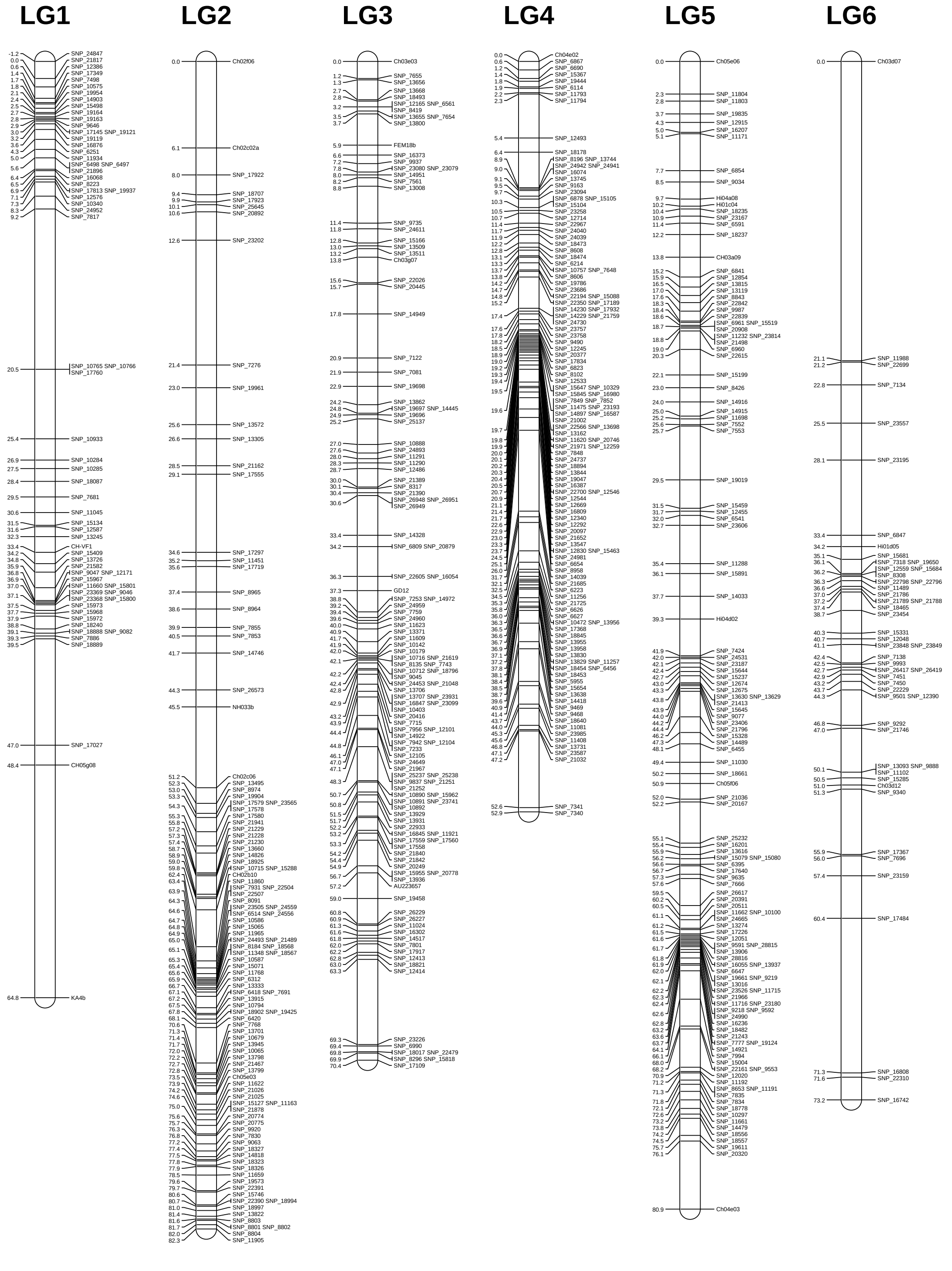

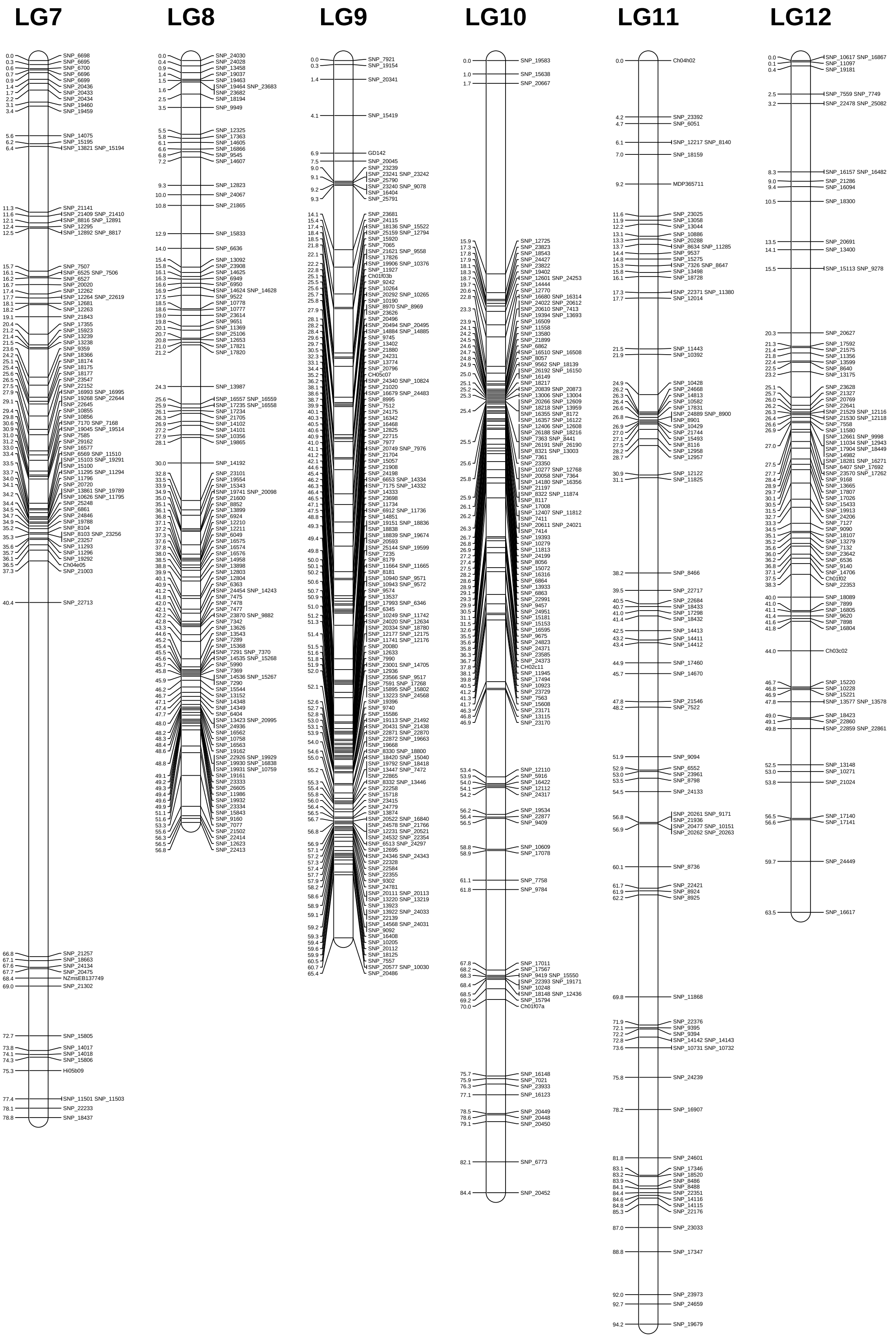
LG7
LG8
LG9
LG10
LG11
LG12

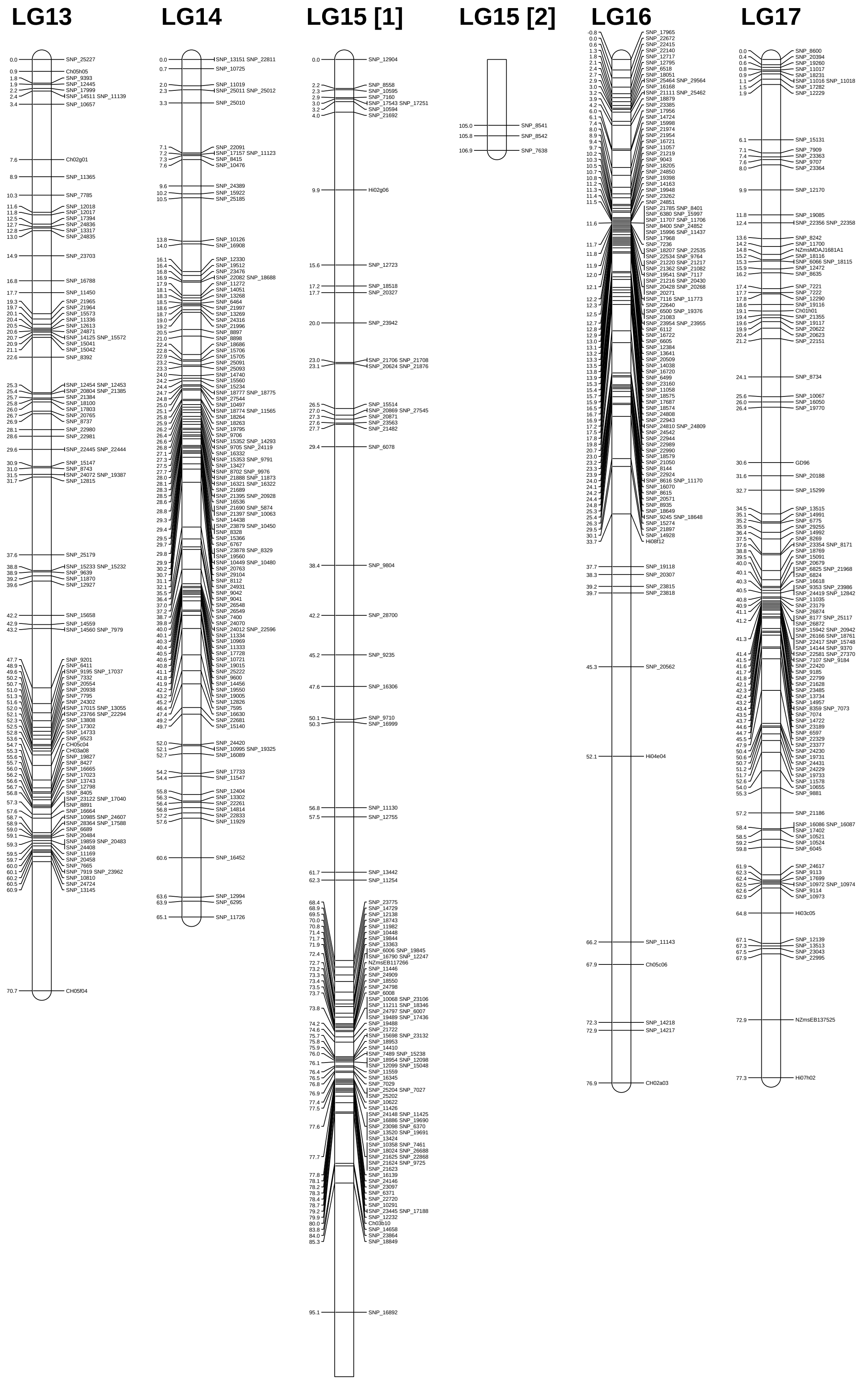
LG13
LG14
LG15 [1]
LG15 [2]
LG16
LG17
